# Treatment of Rhinitis Sicca Anterior with Ectoine Containing Nasal Spray

**DOI:** 10.1155/2014/273219

**Published:** 2014-04-13

**Authors:** Uwe Sonnemann, Olaf Scherner, Nina Werkhäuser

**Affiliations:** ^1^Private Health Centre, Institute for ENT Elmshorn, Hermann-Ehlers-Weg 4, 25337 Elmshorn, Germany; ^2^Bitop AG, Stockumer Street 28, 58453 Witten, Germany

## Abstract

*Objectives.* The safety and efficacy of ectoine nasal spray and ectoine nasal spray with dexpanthenol in the treatment of rhinitis sicca were evaluated in two studies. *Design and Methods.* Two noninterventional observational studies were performed to evaluate the efficacy and safety of a nasal spray containing ectoine (study 1) and ectoine/dexpanthenol (study 2) over a period of two weeks including comparable numbers of patients suffering from rhinitis sicca anterior. Patients and physicians were asked to rate the efficacy in reducing symptoms and the tolerability over the treatment phase. *Results.* The treatment in both studies resulted in a clinical and statistical significant reduction of the main diagnosis parameters, nasal airway obstruction, and crust formation. There was also a significant reduction in the secondary diagnosis parameters in both studies. Importantly, the tolerability was very good. During the whole observational study, neither patients nor doctors stopped the medication due to unwanted effects. *Conclusion.* Rhinitis sicca could be successfully treated with a nasal spray containing ectoine and a nasal spray combining ectoine with dexpanthenol. The combination of both substances led to slight advantages.

## 1. Introduction

Rhinitis sicca or generally speaking dry nose is a rather frequent problem involving many people. The term “dry nose” has not yet been uniformly defined [1]. Otolaryngologists often use the terms “rhinitis sicca” or “dry rhinitis,” although no clear definition exists. Many symptoms during dry nose could be encountered ranging from subjective sensation of the dry nose and itching up to mild burning, nasal obstruction, crusting associated with unpleasant smell, epistaxis, and diminished sense of smell. Rhinitis sicca anterior means a chronic inflammation in the region of the anterior part of the nose, affecting the anterior and caudal septum and/or the corresponding lateral nasal vestibule. Mechanical as well as environmental irritations lead to crust formation. In rare cases, patients suffer from a slight stench due to bacterial colonization of the crust formations. The treatment of rhinitis sicca involves mainly elimination of promoting factors, moistening, sufficient daily drinking amount, cleansing of the crusts, care of the mucosa and inhibition of possible infections, or in rare cases the elimination of overlarge endonasal space [1]. The main treatment for rhinitis sicca consists of humidification of the nose, especially the mucus, focusing in a real wash-out of possible inflammatory triggers and application of a protective layer on the mucus. The market offers a huge number of different devices involving saline, oils, moisturizers, sprays, and ointments for this purpose. Nasal irrigation and nasal saline sprays wash out inflammatory triggers directly [2, 3] and achieve an improvement of mucociliary clearance by improving the ciliary beat frequency [4, 5]. Nasal ointments mostly including glycerol develop a protective moistening effect and protect the nose from water loss [6]. Low concentrated oils also have beneficial effects on nasal ciliary beat frequency [7]. The efficacy of dexpanthenol, the alcohol analog of pantothenic acid, in the treatment of rhinitis sicca is widely spread in the OTC use and has been shown clinically [8]. In addition, use of dexpanthenol has a strong tradition in the treatment of various skin diseases in which dexpanthenol is used as humidifier/moisturizer. Also, use in wound healing has been reported [9]. Besides these different options, patients ask for alternative treatments as the current treatments often leave patients unsatisfied and a demand for other nonpharmacological treatment options exists.

Ectoine is an extremolyte, a compatible solute which is produced by microorganism living under extreme environmental conditions such as extreme salinity or dryness [10]. In those microorganisms, ectoine serves as natural cell protectant [11, 12]. Different in vitro, ex vivo, and in vivo studies have shown that ectoine can be used to protect epithelial tissues and moisturize and reduce inflammations [13–15]. Ectoine acts physically via a mechanism called “preferential exclusion.” In the presence of ectoine, membranes and lipids are protected indirectly: as ectoine is expelled from the surface of proteins and lipids, those are protected by a water shell, thereby increasing the fluidity of membranes and resulting in the preferential formation of the native conformation of proteins [11]. This might stabilize mucous membranes such as lining epithelia of the nose, thereby protecting those cells from invading allergens or pathogens [16]. Recent developments have demonstrated that these cell protective attributes could be transferred into medical devices including ectoine containing creams, nasal sprays, or eye drops which can be used for human use, for example, the treatment of atopic dermatitis, allergic rhinitis, and rhinosinusitis [17–20].

The use of ectoine in a saline based nasal spray could be a useful therapeutic approach for patients suffering from dry nose syndrome. Additionally a combinatory approach could be applied, for example, of ectoine and dexpanthenol. The combined effects of ectoine and/or dexpanthenol are already used in the field of dermatology and promise a useful combination effect for the treatment of rhinitis sicca. By using an ectoine and dexpanthenol nasal spray, the moisturizing and regeneration supporting effects of both compounds could assist a possible healing of ulcers and prevent nasal obstruction in addition to the reduction of primary symptoms.

## 2. Materials and Methods

The current paper describes two prospective, open-label, noninterventional trials (studies 1 and 2). Restricted inclusion of patients study based on the diagnosis of rhinitis sicca and strict adherence to the principle of nonintervention allowed data to be collected for a very unselective patient population. As study designs for both studies were very similar, data are summarized and differences are only outlined where applicable.

### 2.1. Medication

Patients in study 1 were treated with an ectoine containing nasal spray with 0.5% ectoine and further ingredients were sodium chloride, sodium-di-hydrogen-phosphate dihydrate, di-sodium-hydrogen-phosphate, and water.

Patients in study 2 used a 0.5% ectoine nasal spray which contained 1.0% dexpanthenol, sodium chloride, sodium-di-hydrogen-phosphate dihydrate, di-sodium-hydrogen-phosphate, and water.

### 2.2. Treatment and Study Design

Both studies were open for all patients from 18 years on, who were identified by ENT specialist with symptoms of dry nose. Following confirmation of the diagnosis of rhinitis sicca, patients were asked by the ENT specialist whether they were interested to participate in the current trials. Upon signing a patient information and consent form, patients had to attend two more site visits: V2 on day 7 ± 2 and V3 on day 14 ± 2. During the entire treatment period of 2 weeks, patients were asked to use the nasal sprays at least five times daily.

### 2.3. Scoring of Symptoms

Clinical symptoms were assessed on a 12-point scale ranging from 0 (no symptoms) to 12 (very severe symptoms).

During the visits, the physician assessed the main symptoms of nasal obstruction and crusting of the nose as well as the following secondary symptoms: endonasal blood deposits, concomitant pharyngitis, cacosmia, rhinorrhea, exudate viscosity, and turbinate hyperplasia.

On days 3, 6, 9, and 12 after start of the study, patients were asked to document the severity of the following symptoms in a patient diary: nasal obstruction, dryness of the nose, nose bleeding, sore throat, cacosmia, and exudate from the nose. In addition, patients were asked to describe the consistency of exudate on a 12-point scale from 0 (fluid) to 12 (crusted).

## 3. Scoring of Efficacy, Tolerability, and Compliance

Both efficacy and tolerability were assessed by physicians (during V2 and V3) and by the patients (days 3, 6, 9, and 12) on a scale from 0 (very good) to 12 (none/bad).

### 3.1. Statistics

The statistical analysis was carried out with SPSS version 15 (study 1) or 17 (study 2), respectively. Both efficacy and safety analyses were performed on the entire study population. Descriptive statistics were used for a quantitative report of the main study population features. Continuous variables were tested for normal distribution via Kolmogorov-Smirnov test. Further analysis was carried out with the Mann-Whitney* U* test, Wilcoxon test, or Friedman test. The level of significance was set to *P* < 0.05 in all tests. Unavailable data were treated as “missing values” or substituted by the “last value carried forward” method.

## 4. Results

Both studies were conducted in accordance with the Declaration of Helsinki. All investigations were carried out with informed consent of all participants.

**Figure 1:**
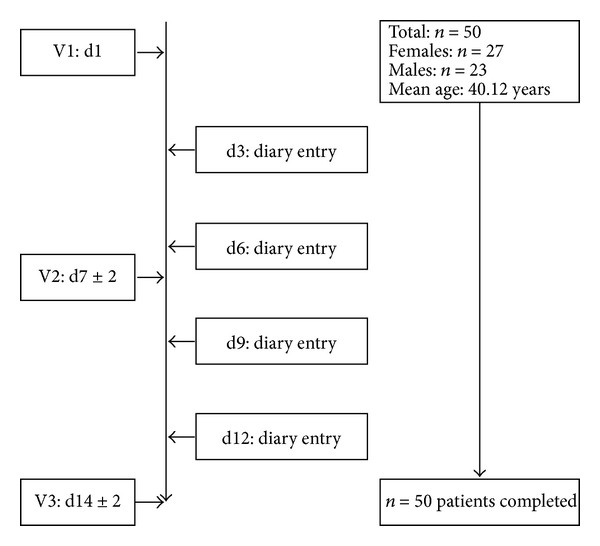
Patient flow and characteristics of demographic data in study 1.

**Figure 2:**
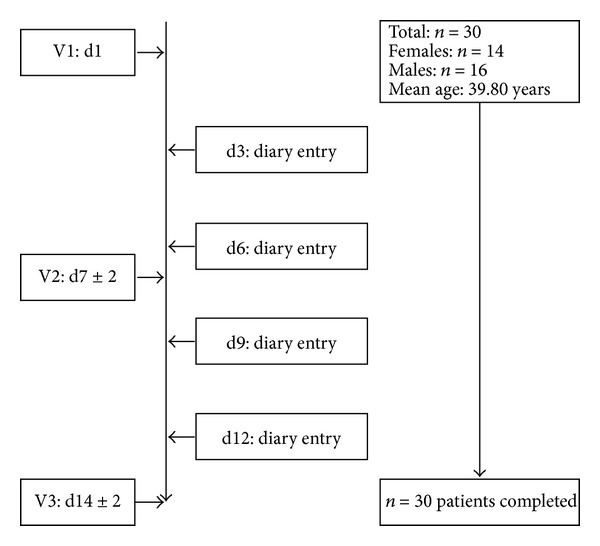
Patient flow and characteristics of demographic data in study 2.

Study 1 was a noninterventional trial taking place from April to July 2008; study 2 took place from March to April 2009. Both studies were carried out at a German ear nose throat (ENT) practice. Distribution and demographics of patients are shown in Figures 1 and 2.

### 4.1. Development of Symptoms

#### 4.1.1. Nasal Obstruction

**Figure 3:**
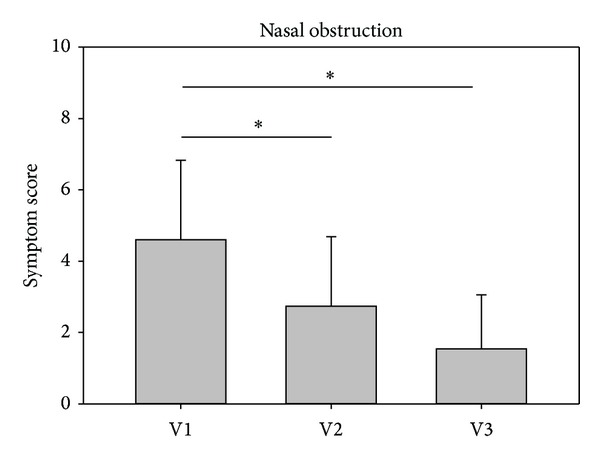
Development of nasal obstruction from V1 to V3 assessed by the investigator (study 1). The asterisks mark a statistical significance; the whiskers mark the standard deviation.

**Figure 4:**
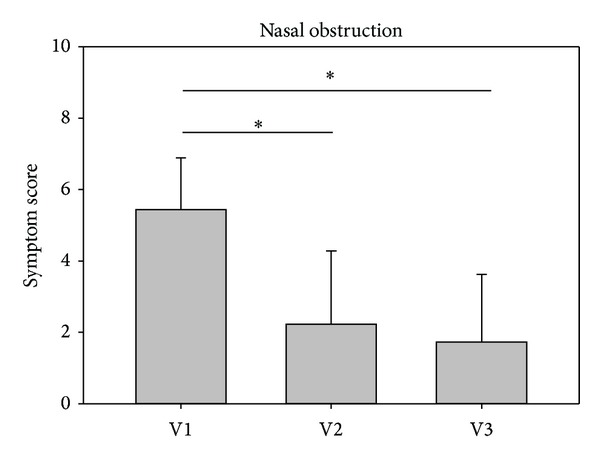
Development of nasal obstruction from V1 to V3 assessed by the investigator (study 2). The asterisks mark a statistical significance; the whiskers mark the standard deviation.

In both studies, the investigators assessment revealed that the symptom nasal obstruction decreased significantly from V1 to V2 as well as further to V3. In study 1, symptom scores decreased from baseline values 4.60 ± 2.23 at V1 to 2.74 ± 1.95 at V2 and then to 1.54 ± 1.52 at V3. Values in study 2 decreased comparably from 5.43 ± 1.46 at V1 to 2.23 ± 2.05 at V2 and further to 1.73 ± 1.89 at V3 (Figures 3 and 4).

Decreases of the symptom nasal obstruction were similar and in accordance with the patients' assessments. Values are listed in Table 1.

#### 4.1.2. Crust Formation/Nasal Dryness

**Figure 5:**
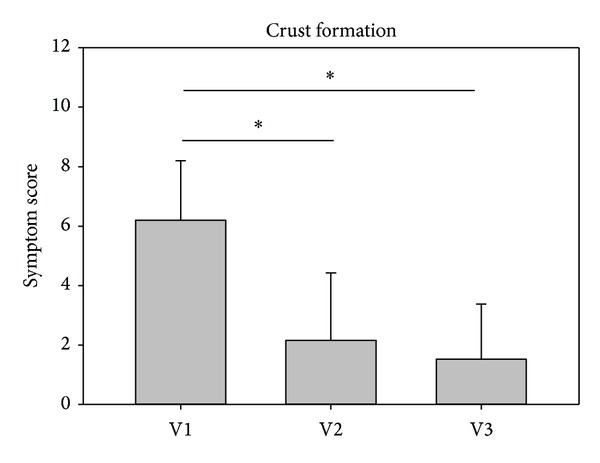
Development of crust formation from visit 1 (V1) to visit 3 (V3) in study 1. *P < 0.001. The asterisks mark a statistical significance; the whiskers mark the standard deviation.

**Figure 6:**
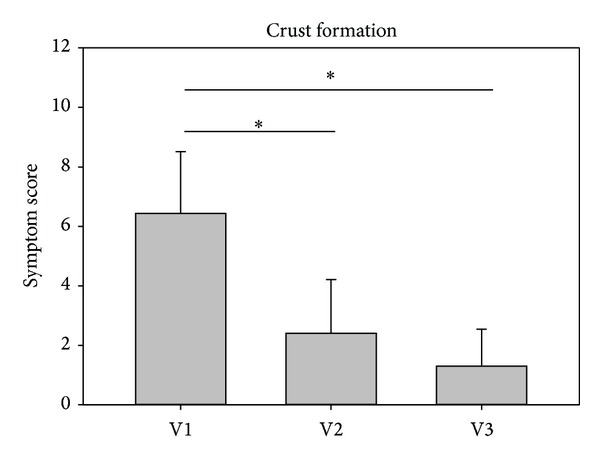
Development of crust formation from visit 1 (V1) to visit 3 (V3) in study 2. *P < 0.001.

The symptom nasal crust formation decreased significantly from V1 to V2 and further to V3 in both studies in the investigators assessment. Values in study 1 decreased from baseline values of 6.20 ± 1.99 to 2.16 ± 2.26 at V2 and further to 1.52 ± 1.85 at V3. Values in study 2 decreased comparably from baseline values of 6.43 ± 2.08 to 2.40 ± 1.81 at V2 and to 1.30 ± 1.24 at V3. Results are depicted in Figures 5 and 6.

Patients evaluated the decrease of the symptom dry nose in a similar way to the physician's assessments as listed in Table 2. The symptom nasal dryness decreased significantly from day 3 to day 12 in both studies.

#### 4.1.3. Secondary Symptom Scores

**Figure 7:**
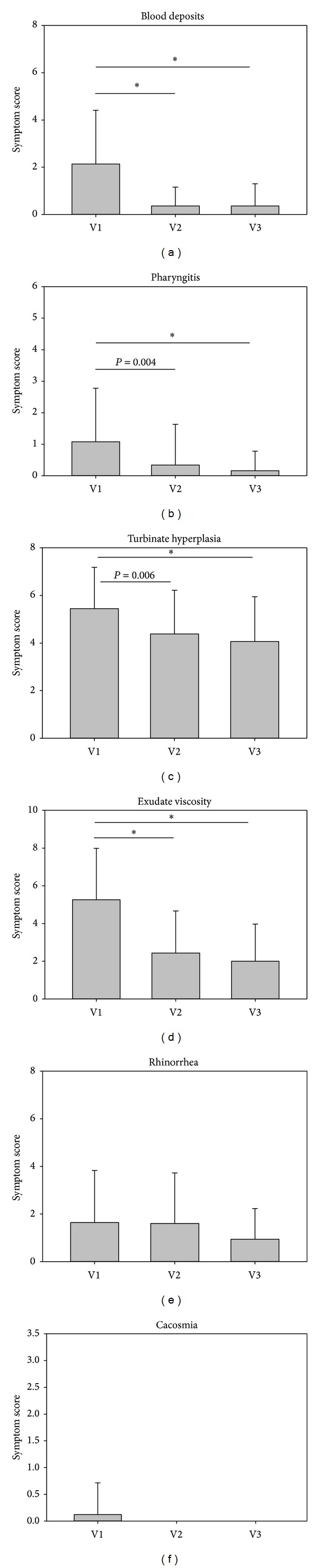
Development of secondary symptoms (ENT evaluation) during study 1. *P < 0.001. The asterisks mark a statistical significance; the whiskers mark the standard deviation.

**Figure 8:**
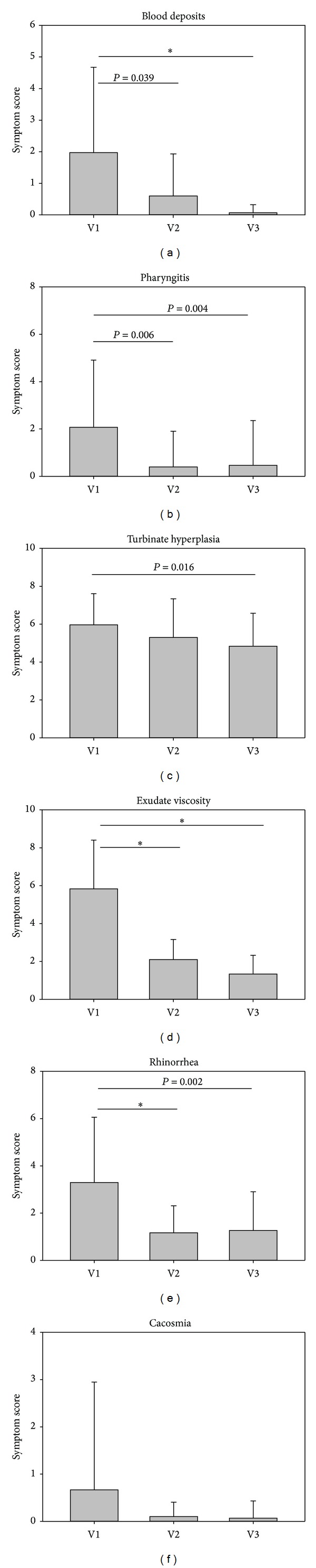
Development of secondary symptoms during study 2. *P < 0.001.

In addition to the symptoms nasal obstruction and crust formation/nasal dryness, further symptoms were assessed by both investigators and patients. As depicted in Figures 7 and 8, there was a similarity between the results of both studies in the investigators assessment. The symptoms blood deposits, pharyngitis, turbinate hyperplasia, and exudate viscosity improved significantly from baseline values at V1 to the final visit V3. The symptom rhinorrhea only improved significantly in study 2, whereas decreases in this symptom were nonsignificant in study 1. As only very few patients complained about the symptom cacosmia (*n* = 2 in study 1 and *n* = 5 in study 2), decreases in this symptom were negligible.

Patients' scores of secondary symptom evaluation are listed in Tables 3 and 4. In study 1, the symptom rhinorrhea improved significantly from d3 to d12. In study 2, nose bleeding, rhinorrhea, cacosmia, and exudate viscosity improved significantly over this time frame.

## 5. Efficacy, Tolerability, and Compliance

**Figure 9:**
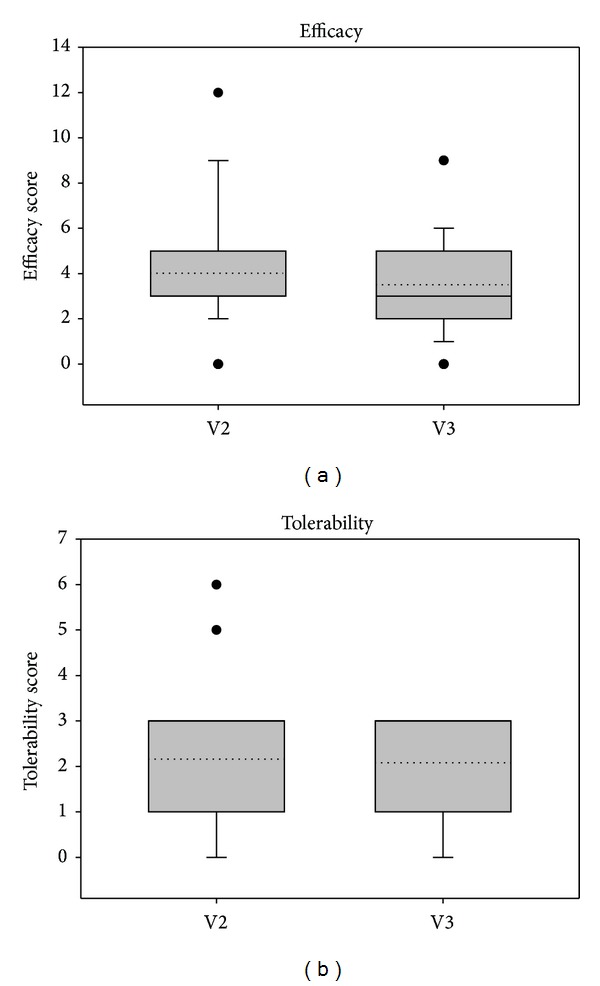
Judgment of efficacy and tolerability according to the physician's assessment in study 1.

**Figure 10:**
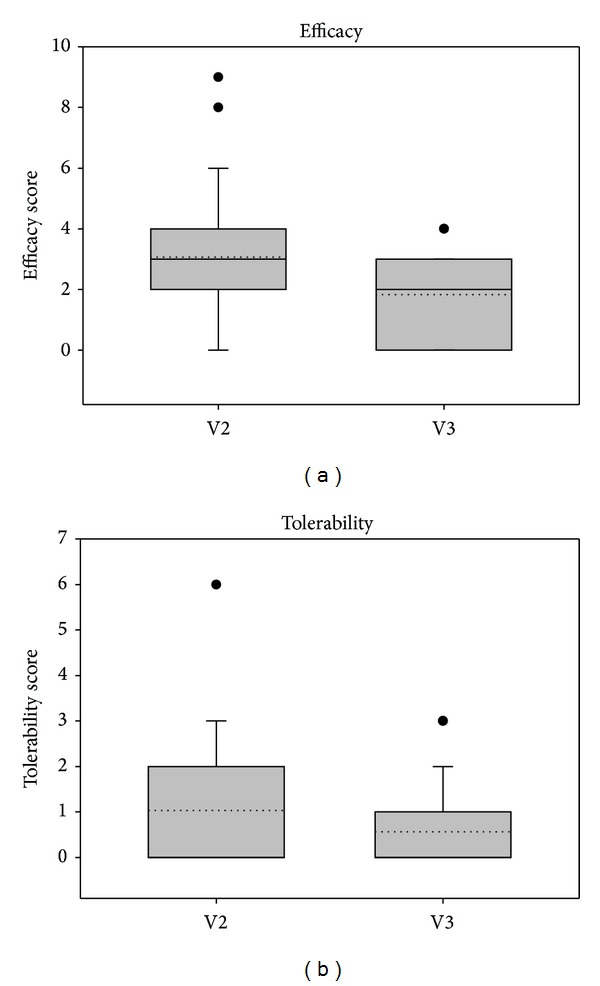
Judgment of efficacy and tolerability according to the physician's assessment in study 2.

The physician judged both efficacy and tolerability of treatment after 7 days (V2) and after 14 days (V3). As shown in Figures 9 and 10, ectoine nasal spray treatment was considered to be both efficient and well tolerable. Mean values for efficacy at V3 were 3.5 ± 2.06 (study 1) and 1.83 ± 1.39 (study 2) meaning good to very good efficacy. Mean values for tolerability were 2.08 ± 1.21 (study 1) and 0.57 ± 0.97 (study 2), which also means good to very good tolerability.

**Figure 11:**
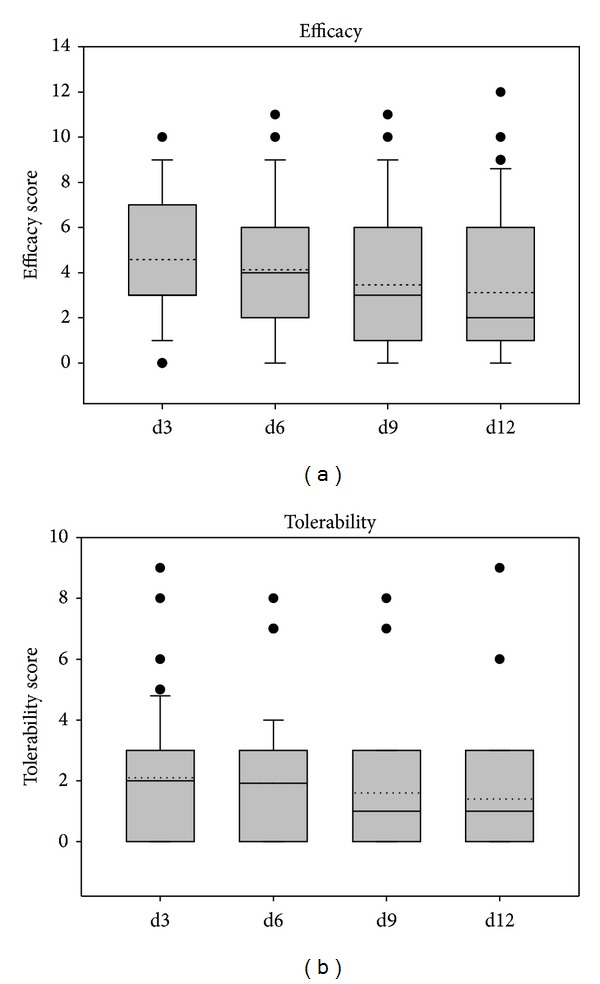
Judgment of efficacy and tolerability according to the patients' assessment in study 1.

**Figure 12:**
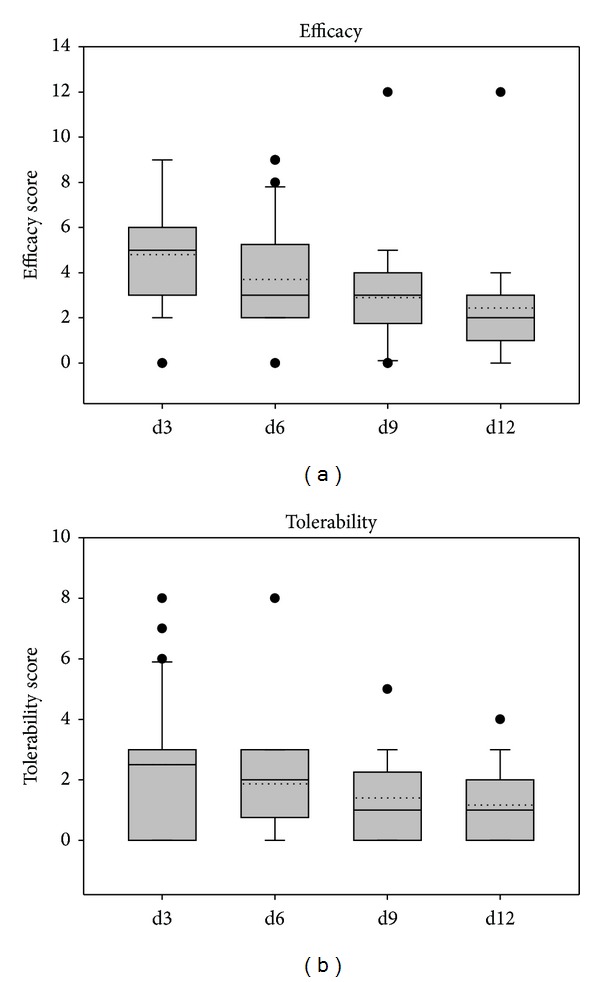
Judgment of efficacy and tolerability according to the patients' assessment in study 2.

Patients' assessments of tolerability and efficacy of treatment are depicted in Figures 11 and 12. Mean efficacy values were 3.12 ± 3.08 at day 12 of treatment in study 1 and 2.43 ± 2.24 in study 2 corresponding to good efficacy. Tolerability was judged as very good in both studies with mean values on day 12 of 1.40 ± 1.80 in study 1 and 1.17 ± 1.21 in study 2.

### 5.1. Adverse Events (AEs)

In study 1, no AE occurred. In study 2, 1 AE occurred (acute rhinitis). The correlation with the treatment was judged as unlikely by the investigator. No SAE occurred in either of the two studies.

## 6. Conclusions

The aim of these observational studies was to gain insight into the tolerability and the extent to which the treatments influenced the severity of the patients' symptoms. An ectoine nasal spray (study 1) or an ectoine and dexpanthenol nasal spray (study 2) was tested in patients with rhinitis sicca under practical conditions. A total of 80 patients (50 patients in study 1, 30 patients in study 2) with a wide variety of disease severities participated in this postmarketing surveillance study. However, the potential flaw of these studies is their noncontrolled character, the missing randomization, or placebo control. Therefore the evidence grade of the results needs to be reduced at least to IIb.

Both nasal spray formulations showed a good tolerability and safety in the studies. No drop-out was recorded. The studies showed a significant decrease of the main symptoms nasal obstruction and crust formation from V1 to V2 as well as further to V3. The decrease of nasal obstructions assessed by the physicians was confirmed by patients in both studies, with a stronger decrease of symptoms assessed by the physicians. This is likely to be due to the timing of the patient's diary, as this was started at day three of treatment, when the first positive effect of the respective treatments had occurred already.

Apart from main symptoms, the secondary symptom scores also decreased similarly in both studies. In the investigators assessment, the symptoms blood deposits, pharyngitis, turbinate hyperplasia, and exudate viscosity improved significantly from starting values at V1 to the final visit V3. Differences in symptom reduction between both studies occurred only with respect to rhinorrhea. Treatment with the nasal spray with ectoine only did not lead to a significant improvement of rhinorrhea, whereas the improvement in with the ectoine nasal spray alone was not statistically significant. The degree of symptom reduction in the main parameter nasal obstruction seemed to be reduced more efficiently in the study with the combined nasal spray, as the score started with a higher value and dropped faster and to a higher degree as in the study with the ectoine nasal spray. In the patient assessment of study 2 symptom improvement was significant for nose bleeding, exudate viscosity, rhinorrhea, and cacosmia, whereas the patient assessment in study 1 showed only a significant reduction of the symptoms in nose bleeding. It can be mentioned that only a few patients in both studies suffered from cacosmia and the decreases in these symptoms were negligible for both of them.

As a summary, the ectoine nasal spray achieved in study 1 treatment success similar to that of the combination of ectoine and dexpanthenol in study 2 with respect to the main symptom scores of both studies, crust formation and nasal obstruction. Differences in treatment effect between both nasal sprays and studies were observable in the secondary parameters, both in physicians and in patients assessment, tending towards an additional effect if ectoine and dexpanthenol are combined in one product compared to ectoine alone. Both natural nonpharmacological nasal sprays showed efficacy in treatment of rhinitis sicca, which is comparable to the reported outcome for other products [21]. Data from preclinical studies also support the combination of ectoine and dexpanthenol (data not shown).

The mode of action and subsequent effect in treatment of rhinitis sicca of dexpanthenol is understood from the literature [22]. The treatment effects of the ectoine nasal spray can be attributed to its physical action. By increasing the fluidity of the nasal epithelia, the barrier function of this membrane is increased, therefore inhibiting the potential loss of water. Experiments with ectoine on biological and artificial membranes support this thesis further, including the reduction of mechanical stress induced membrane damage [15–17]. The additional hydrating effect of ectoine is well described in the literature [15, 23, 24] as well as the capacity of reduction of inflammation in skin and respiratory epithelium [14, 17, 18, 25].

Taken together, rhinitis sicca anterior or dry nose could be successfully treated with an ectoine containing nasal spray. Therefore an interesting option of a new and safe nonpharmacological treatment of rhinitis sicca will be available in the future. The addition of the well-known and accepted dexpanthenol did not enhance the treatment regarding the major symptoms scores. The decrease of symptom over the 14-day treatment period was more pronounced for the combination of ectoine and dexpanthenol, but this difference was not significant. Slightly better improvement in different secondary symptoms revealed synergistic characteristics of the two substances in combination when compared to the ectoine nasal spray alone. However, these findings came from two independent noncontrolled trials. Therefore additional controlled trials are suggested to further prove the efficacy of ectoine nasal spray with or without dexpanthenol.

## Figures and Tables

**Table 1:** Development of nasal obstruction assessed by patients on days 3, 6, 9, and 12 following treatment with ectoine nasal spray.

	d3	d6	d9	d12	*P* (d3 versus d12)
Study 1	2.74 ± 2.31	2.52 ± 2.18	2.10 ± 2.00	1.64 ± 1.68	<0.001
Study 2	3.67 ± 2.28	2.80 ± 1.99	2.33 ± 1.65	1.87 ± 1.33	<0.001

**Table 2:** Development of nasal dryness assessed by patients on days 3, 6, 9, and 12 following treatment with ectoine nasal spray.

	d3	d6	d9	d12	*P* (d3 versus d12)
Study 1	4.64 ± 2.40	3.76 ± 2.53	2.90 ± 2.48	2.42 ± 2.37	<0.001
Study 2	4.43 ± 2.49	3.30 ± 2.12	2.33 ± 1.97	1.83 ± 1.49	<0.001

**Table 3:** Patients' assessments of secondary symptom scores at days 3, 6, 9, and 12 following treatment start of study 1.

Symptoms	d3	d6	d9	d12	*P* value (d3 versus d12)
Nose bleeding	0.36 ± 0.85	0.46 ± 1.07	0.36 ± 0.85	0.30 ± 0.74	0.787
Pharyngitis	0.64 ± 1.77	0.62 ± 1.74	0.46 ± 1.20	0.42 ± 1.13	0.754
Exudate viscosity	3.36 ± 3.50	2.90 ± 3.03	2.38 ± 2.86	2.40 ± 2.93	0.108
Rhinorrhea	2.44 ± 2.16	2.10 ± 2.14	1.74 ± 1.88	1.42 ± 1.54	0.021
Cacosmia	0.60 ± 1.53	0.48 ± 1.31	0.42 ± 1.57	0.26 ± 0.69	0.388

**Table 4:** Patients' assessments of secondary symptom scores at days 3, 6, 9, and 12 following treatment start of study 2.

Symptoms	d3	d6	d9	d12	*P* value (d3 versus d12)
Nose bleeding	0.70 ± 1.60	0.73 ± 1.72	0.50 ± 1.01	0.17 ± 0.59	0.0019
Pharyngitis	1.07 ± 2.26	0.67 ± 1.63	0.57 ± 1.19	0.60 ± 1.00	0.719
Exudate viscosity	2.67 ± 2.38	2.50 ± 2.64	2.10 ± 2.23	1.67 ± 2.19	0.027
Rhinorrhea	2.57 ± 1.99	2.00 ± 1.72	1.90 ± 1.83	1.57 ± 1.45	0.025
Cacosmia	0.90 ± 2.26	0.70 ± 1.82	0.33 ± 0.99	0.30 ± 0.99	0.005
